# Impact of non-axillary sentinel node biopsy on staging and treatment of breast cancer patients

**DOI:** 10.1038/sj.bjc.6600359

**Published:** 2002-09-23

**Authors:** P J Tanis, O E Nieweg, R A Valdés Olmos, J L Peterse, E J Th Rutgers, C A Hoefnagel, B B R Kroon

**Affiliations:** Department of Surgery, The Netherlands Cancer Institute/Antoni van Leeuwenhoek Hospital, Amsterdam, The Netherlands; Department of Nuclear Medicine, The Netherlands Cancer Institute/Antoni van Leeuwenhoek Hospital, Amsterdam, The Netherlands; Department of Pathology, The Netherlands Cancer Institute/Antoni van Leeuwenhoek Hospital, Amsterdam, The Netherlands

**Keywords:** breast cancer, lymphoscintigraphy, sentinel node, staging

## Abstract

The purpose of this study was to evaluate the occurrence of lymphatic drainage to non-axillary sentinel nodes and to determine the implications of this phenomenon. A total of 549 breast cancer patients underwent lymphoscintigraphy after intratumoural injection of ^99m^Tc-nanocolloid. The sentinel node was intraoperatively identified with the aid of intratumoural administered patent blue dye and a gamma-ray detection probe. Histopathological examination of sentinel nodes included step-sectioning at six levels and immunohistochemical staining. A sentinel node outside level I or II of the axilla was found in 149 patients (27%): internal mammary sentinel nodes in 86 patients, other non-axillary sentinel nodes in 44 and both internal mammary and other non-axillary sentinel nodes in nineteen patients. The intra-operative identification rate was 80%. Internal mammary metastases were found in seventeen patients and metastases in other non-axillary sentinel nodes in ten patients. Staging improved in 13% of patients with non-axillary sentinel lymph nodes and their treatment strategy was changed in 17%. A small proportion of clinically node negative breast cancer patients can be staged more precisely by biopsy of sentinel nodes outside level I and II of the axilla, resulting in additional decision criteria for postoperative regional or systemic therapy.

*British Journal of Cancer* (2002) **87**, 705–710. doi:10.1038/sj.bjc.6600359 www.bjcancer.com

© 2002 Cancer Research UK

## 

Lymph nodes in the internal mammary chain are known to be a potential site of metastases from breast cancer. Extended radical mastectomy has been used to obtain information about the status of these lymph nodes. The internal mammary node status is an independent prognostic factor for survival (Cody III and Urban, 1995). However, treatment of internal mammary lymph nodes is subject of debate. The value of elective radiotherapy to these nodes has never been convincingly shown and is, in fact, the subject of ongoing randomised studies in Europe and in Canada. Elective dissection of internal mammary nodes is not done.

Besides the axilla and internal mammary chain, breast cancer spreads to interpectoral nodes (Rotter's nodes) in 3–14% of the patients (Cody III *et al*, 1984; Dixon *et al*, 1993; Bale *et al*, 1999). A Rotter's node is the only site of metastatic involvement in a small number of patients (Cody III *et al*, 1984; Dixon *et al*, 1993; Bale *et al*, 1999). The importance of intraparenchymal and supra- and infraclavicular lymph nodes in breast cancer is unknown and involvement of these lymph nodes has been described only incidentally (Hyman and Abellera, 1974; Veronesi *et al*, 1987; Nathanson and Wachna, 1999; Rull *et al*, 1999).

The technique of lymphatic mapping visualises the lymph nodes that directly receive lymph from a primary breast carcinoma. These ‘sentinel’ nodes can be harvested in the axilla and reflect the tumour-status of the entire axilla with a high sensitivity (Cox *et al*, 1998; Doting *et al*, 2000; Giuliano *et al*, 2000). The purpose of this study was to determine the incidence of lymphatic drainage and metastatic spread to sentinel nodes outside the axilla in early stage breast cancer and the impact of biopsy of these lymph nodes on staging and treatment strategy.

## PATIENTS AND METHODS

Between January 1997 and July 2001, 549 patients with a clinical T1-3N0 breast cancer underwent sentinel node biopsy in The Netherlands Cancer Institute. The mean age was 56 years (range 27–91 years). Six patients had bilateral tumours. Pathologic proof of breast cancer was routinely obtained. The primary lesion was still present in all patients. Seventy-one per cent of the patients underwent wide local excision as part of breast-conserving treatment and the remaining patients underwent mastectomy. The mean histological tumour diameter was 1.9 cm (range 0.2–8.0 cm) with a pT1 stage in 82.9%, pT2 in 16.4% and pT3 in 0.7%. Definitive pathological examination showed ductal carcinoma *in situ* without invasion in twelve patients. The first 82 patients were part of a learning phase study with confirmatory axillary lymph node dissection (Doting *et al*, 2000). In the subsequent patients, axillary clearance was omitted in case of a tumour-negative axillary sentinel node.

### Sentinel node biopsy

The day before surgery, ^99m^Tc-Nanocolloid (Amersham Cygne, Eindhoven, the Netherlands) was injected into the tumour in a mean volume of 0.2 ml and with a mean radioactive dose of 104 Mbq (range 42–159 Mbq). Subsequently, dynamic and static anterior and prone lateral (hanging breast) images were obtained (Valdés Olmos *et al*, 2000). A hot spot was considered to be a sentinel node if an afferent lymphatic channel was visualised, the hot spot was the first one seen in a sequential pattern, the hot spot was the only one in a particular lymph node basin or when a combination of criteria was present. Intramammary, paramammary (Gerota) and interpectoral sentinel nodes were defined as interval nodes, because of their location on the drainage route to the axilla or internal mammary chain (Caplan, 1975). The location of a sentinel node was marked on the skin. The following day, patent blue dye (Blue Patenté V, Laboratoire Guerbet, Aulnay-sous-Bois, France) in a volume of 1.0 ml was injected into the tumour. The sentinel node was identified and harvested after careful dissection of blue lymphatic vessels and detection of radioactivity with a gamma ray detection probe (Neoprobe®, Johnson & Johnson Medical, Hamburg, Germany). Internal mammary sentinel nodes were explored through a small transverse incision over the intercostal space concerned. After splitting the pectoral muscle fibres and dividing the intercostal muscles, radioactive lymph nodes with or without blue discoloration were dissected from the internal mammary vessels and parietal pleura. It is our policy to perform a complete axillary lymph node dissection if no sentinel node is identified when exploring the axilla unless one is identified elsewhere. Sentinel nodes were formalin-fixated, bisected, paraffin-embedded and cut at a minimum of six levels at 50 to 100 μg intervals. Paraffin sections were stained with haematoxylin-eosin and immunohistochemistry (CAM5.2, Becton Dickinson, San Jose, CA, USA).

### Postoperative treatment

Traditionally, the indication for radiotherapy of the internal mammary lymph nodes at our institution was a positive axillary lymph node irrespective of tumour location and size unless the patient was enrolled in an ongoing EORTC trial (no. 22922). Currently, a patient will receive radiotherapy to the parasternal area if an excised internal mammary sentinel lymph node is tumour-positive. Adjuvant systemic treatment is generally given to patients with lymph node metastasis over 2 mm in size. The Dutch national guidelines recommend that node-negative patients with a tumour larger than 3.0 cm and patients with a high-grade tumour between 1.0 and 3.0 cm in diameter (grade III or mitotic activity index of greater than 10 per ten high power fields) receive adjuvant chemotherapy or hormonal treatment (Bontenbal *et al*, 2000).

### Statistical analysis

The chi square test was used to compare the incidence of lymph node metastasis between subgroups of patients. Values of *P*⩽0.05 were considered to be statistically significant. Statistical analyses were performed with Statistical Package for the Social Sciences software (SPSS, Chicago, IL, USA).

## RESULTS

Sentinel nodes outside level I and II of the axilla were seen on the lymphoscintigraphy images in 147 patients and in an additional two patients intraoperatively using patent blue dye. Therefore, the incidence was 27% (149 out of 549 patients). The lymphatic drainage patterns are displayed in Table 1

**Table 1:**
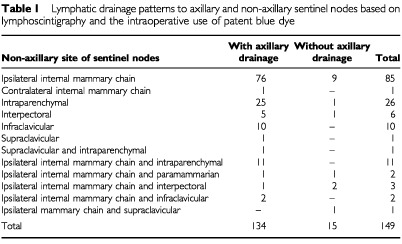
Lymphatic drainage patterns to axillary and non-axillary sentinel nodes based on lymphoscintigraphy and the intraoperative use of patent blue dye

. Drainage to the ipsilateral internal mammary sentinel nodes was observed in 104 patients (19%). A contralateral internal mammary sentinel node was seen in one additional patient. Interval sentinel nodes were encountered in 49 patients: intramammary sentinel nodes in 38, paramammary nodes in two and interpectoral sentinel nodes in nine patients. Twelve patients had sentinel nodes in the infraclavicular region and three in the supraclavicular region.

At least one non-axillary sentinel node was excised in 128 out of 149 patients (86%). The results of the surgical identification of sentinel nodes outside level I and II of the axilla are displayed in Table 2

**Table 2:**
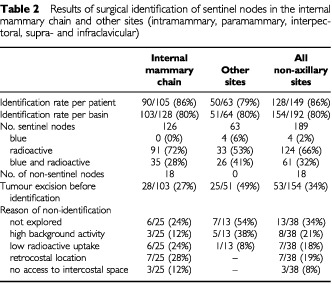
Results of surgical identification of sentinel nodes in the internal mammary chain and other sites (intramammary, paramammary, interpectoral, supra- and infraclavicular)

. High background radioactivity from tracer remaining at the injection site necessitated segmental excision of the primary lesion before the sentinel node could be identified with the probe in 53 patients (34%). In eight patients, the extra-axillary sentinel node could not be identified because of high background radioactivity that remained after excision of the primary lesion (Table 2). Sentinel nodes were either blue, radioactive or both. A learning phase for biopsy of internal mammary chain nodes was observed: the identification rate was 70% in the first 30 procedures and 84% in the following procedures.

Metastases were found in 26 of the 128 patients (20%) who underwent extra-axillary sentinel node excision. Metastases were found in the internal mammary chain in 16 patients, in the breast parenchyma in five, between the pectoral muscles in one, in both the internal mammary chain and interpectoral fossa in one, in the infraclavicular fossa in one, in a paramammary sentinel node in one and in both the supraclavicular fossa and breast parenchyma in another patient.

Metastases in the internal mammary chain were found in two out of 132 grade I tumours (2%), in ten out of 240 grade II tumours (4%), in three out of 132 grade III tumours (2%) and in two out of 51 tumours with an unknown differentiation grade. The incidence of internal mammary node metastases was 3% in both T1 and T2 primary tumours. Internal mammary node metastases were found by immunohistochemistry only in one T1G2 tumour, in two T1G3 tumours and in two T2G2 tumours. The relation between the tumour-status of the internal mammary chain sentinel nodes, the axillary status and the primary tumour site is described in Table 3

**Table 3:**
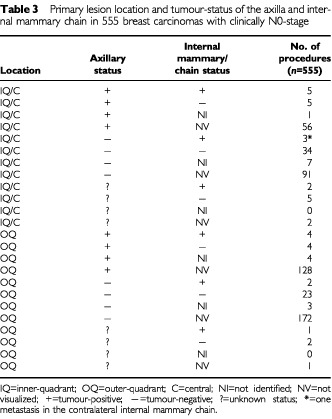
Primary lesion location and tumour-status of the axilla and internal mammary chain in 555 breast carcinomas with clinically N0-stage

. Although there was a higher incidence of internal mammary node metastases in inner quadrant and central tumours in comparison to outer quadrant lesions (5 *vs* 2%), this difference is not significant (*P*=0.073).

Axillary node-positive patients had a higher rate of internal mammary node involvement than axillary node-negative patients (4 *vs* 1%, *P*=0.042). This difference was more pronounced between the subgroups of axillary node-positive and node-negative patients in whom an internal mammary node was excised (50 *vs* 8%, *P*<0.001).

The non-axillary sentinel node was the only tumour-positive lymph node in eleven out of 206 patients (5%) with tumour-positive sentinel nodes. The location of the metastases, the characteristics of the primary tumour and the adjuvant therapy in these eleven patents are described in Table 4

**Table 4:**
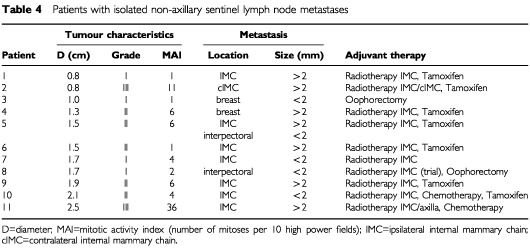
Patients with isolated non-axillary sentinel lymph node metastases

.

Of the total of 17 patients with internal mammary node metastasis, eight (47%) had no axillary involvement. Four out of ten patients (40%) with other non-axillary lymph node metastases also had a tumour-negative axilla. Considering that one patient had extra-axillary metastases at more than one site, the non-axillary sentinel node status had an impact on staging (N1 or N3 instead of N0) in eleven patients with isolated non-axillary sentinel node metastases. These metastases were found by routine H&E staining in 10 patients and by immunohistochemistry only in one patient. Nine patients with metastases both in the internal mammary sentinel node and in the axilla were upstaged from N1 to N3. Overall, N-staging was changed in 20 out of 149 patients (13%).

Management was modified in several respects in 26 out of 149 patients (17%), which comprises 5% of the whole sample population. Internal mammary chain irradiation was given to seven of the 149 patients (5%) which they would not have received if the prior guidelines had been followed. Internal mammary chain irradiation was not given to nine patients (6%) who otherwise would have received such treatment. Eight patients (5%) received adjuvant chemotherapy or hormone treatment only because of isolated non-axillary sentinel lymph node metastases (Table 4). The finding of an extra-axillary sentinel node prevented axillary lymph node dissection in ten patients (7%) without a blue or radioactive lymph node in the axilla. No axillary recurrence has occurred in these ten patients during a median follow-up of 19 months (range 2–29 months).

## DISCUSSION

Sentinel nodes were located outside levels I and II of the axilla in 27% of the patients. Their removal led to a change in the management in 17% of them, although this percentage depends on the protocol for adjuvant therapy that is used (Love, 2002).

Successful visualisation and identification of non-axillary sentinel nodes requires certain specific elements of the technique of lymphatic mapping. Preoperative lymphoscintigraphy is indispensable to detect non-axillary sentinel nodes. An intraparenchymal tracer administration is essential because intradermal or subdermal injection will rarely visualise drainage to the internal mammary chain (Shen *et al*, 2001; Tanis *et al*, 2001). Intratumoural injection with a small volume is preferred to peritumoural injection, because it limits the extent of the diffusion zone at the injection site (Valdés Olmos *et al*, 2000). A single intralesional tracer administration improves imaging of nearby intraparenchymal sentinel nodes and enables elimination of background radioactivity by tumour excision to facilitate subsequent probe identification of such nodes (Table 2).

Harvesting lymph nodes outside the axilla is often a technical challenging procedure. Internal mammary nodes are generally not more than a few millimeters in size and are sometimes located behind a rib. The probe is difficult to handle in the narrow intercostal space. The intercostal space is sometimes inaccessible due to bony deformation or location of the ribs close to each other. A mere 28% of the internal mammary nodes contained blue dye, which can be explained by the fact that internal mammary chain nodes were mostly explored at the end of the procedure at which time the blue dye may have passed the sentinel node. Additionally, the supply of blue dye was removed in 27% because the primary tumour was excised before the sentinel was found. Increasing experience of the surgeons and possibly the increase in radioactivity dose and colloid particle concentration resulted in the improvement of identification from 70 to 84% (Valdés Olmos *et al*, 2001). Morbidity was limited to an occasional injury to the internal mammary artery or pleura. In a few patients, a separate incision of a few centimetres was necessary. Long-term sequelae were not encountered.

Some of the technical aspects outlined above can explain the large differences in the reported incidence of non-axillary sentinel nodes (Uren *et al*, 1995; Roumen *et al*, 1997; Borgstein *et al*, 1998; Chatterjee *et al*, 1998; O'Hea *et al*, 1998; Reuhl *et al*, 1998; Rubio *et al*, 1998; Snider *et al*, 1998; Hill *et al*, 1999; Liberman *et al*, 1999; Miner *et al*, 1999; Imoto *et al*, 2000). Three studies concern results of biopsy of non-axillary sentinel nodes in a substantial number of patients (Krag *et al*, 1998; Johnson *et al*, 2000; Zurrida *et al*, 2000). None of these investigators mentioned that the pursuit of sentinel nodes outside the axilla has an impact on the way patients are managed. van der Ent *et al* (2001) reported drainage to the internal mammary chain in 65 out of 256 patients (25%). Sentinel nodes at this site could be harvested in 41 patients (63%). These nodes contained metastasis in eleven patients (27%), resulting in a change of the subsequent management. The incidence of internal mammary sentinel nodes in the present study is comparable to Van der Ent's observations. The better identification rate in the present study (86 *vs* 63%) may be due to differences in the technique.

A result of our study design is that the sensitivity cannot be determined because no completion internal mammary lymph node dissection was done. Noguchi *et al* (2000) routinely performed biopsy of internal mammary nodes in the first and second intercostal spaces irrespective of the lymphoscintigraphy results and concluded that lymphatic mapping is an insensitive technique for identifying metastases to these nodes. The limited sensitivity in that study may be explained by the low visualisation rate of internal mammary sentinel nodes: 9% *vs* twice as many as in our study.

Our study shows that chasing extra-axillary sentinel nodes improves staging in 13% of the patients. This has therapeutic implications, because it is generally accepted that metastases in these lymph nodes are of prognostic significance. The overall survival rates of patients with isolated internal mammary chain metastases are similar to those with isolated axillary lymph node involvement. The lowest survival rates are observed in patients with both axillary and internal mammary node metastases (Handley, 1975; Urban, 1978; Veronesi *et al*, 1983). Whether these ramifications of improved staging result in a survival benefit is unknown. Lacour *et al* (1983) and Meier *et al* (1989) performed randomised studies of extended radical mastectomy *vs* radical mastectomy and found no survival difference. Randomised trials of post-mastectomy radiation encompassing the internal mammary nodes did not result in an improvement in overall survival either (Fisher *et al*, 1970; Palmer and Ribeiro, 1985; Høst *et al*, 1986; Veronesi *et al*, 1986; Arriagada *et al*, 1996). Obfuscating factors of such studies can be the use of adjuvant systemic treatment and the fact that internal mammary nodes may be situated in the breast irradiation field (Freedman *et al*, 2000). Another disadvantage of these studies is dilution of the potential benefit of the therapy, because the patients who indeed have lymph node metastases are a minority within these populations. This was illustrated by Lacour *et al* (1976) and Meier *et al* (1989) who showed a survival benefit in subgroups of patients with an expected higher incidence of internal mammary node metastasis.

Traditionally, radiotherapy to the internal mammary chain is often given in patients with a primary tumour over a certain size or in a medical quadrant, or in the presence of an involved axilla. Looking at the primary lesion site and the tumour-status of the axilla is a fairly crude way to select patients who may have internal mammary lymph node metastases (Table 3). Biopsy of internal mammary sentinel nodes allows one to identify patients who indeed have metastatic disease in these nodes. Surgical treatment of internal mammary lymph node metastases seems excessive in this era of conservative surgical treatment of breast cancer but radiotherapy could be considered. Common sense suggests that radiotherapy is of no value in case of a tumour-free internal mammary sentinel node, even in the presence of a large primary tumour in an inner quadrant with axillary node metastases.

What about patients who do have metastatic disease in an internal mammary sentinel node? Radiotherapists and medical oncologists should modify their consensus protocols for radiotherapy and adjuvant systemic treatment to include these patients. Incorporating the tumour-status of sentinel nodes outside the axilla in the management may lead to better patient selection and improved regional control and survival. One would like to examine these issues in randomised trials but such trials will be difficult to conduct. For instance, an informed patient with a tumour-positive internal mammary sentinel node might be hesitant to accept the risk of being excluded from adjuvant radiotherapy and systemic treatment.

How should we manage patients with a sentinel node outside the axilla but without an axillary hot spot on the lymphoscintigraphy images? Twenty-three such patients were encountered. Our policy to explore the axilla and to look for blue or radioactive lymph nodes resulted in the retrieval of a sentinel node in eight of these patients. In 10 of the 15 remaining patients, we refrained from an axillary lymph node dissection, which would have been performed in the absence of an extra-axillary sentinel node. One may argue that tracer uptake in an axillary sentinel node was blocked by massive tumour infiltration. The risk of overlooking such a metastasis was reduced by preoperative ultrasound of the axilla and careful intraoperative palpation of the biopsy wound (Tanis *et al*, 2000). No axillary recurrence was seen during follow-up of these ten patients.

The more accurate staging that is the result from lymphatic mapping may have repercussions for the TNM staging system. For instance, an internal mammary lymph node metastasis is currently classified as N3. A metastasis in an internal mammary *sentinel* node is detected at an early stage and carries a prognosis that is probably not much worse than is true for an involved sentinel node in the axilla, which is classified as N1. Another subject of debate is the clinical relevance of micrometastasis, especially if detected by techniques like step sectioning or immunohistochemical staining (Yarbro *et al*, 1999). Six of 17 patients (35%) had an internal mammary node metastasis smaller than 2 mm. A future evaluation of the TNM system needs to address these issues.

## CONCLUSIONS

Lymphatic drainage to sentinel nodes outside levels I and II of the axilla was found in 27% of the patients. Such nodes contain relevant staging information. Specific technical elements are essential to identify and harvest these lymph nodes. Sentinel nodes outside the axilla could be surgically identified in the majority of the patients and contained metastasis in 20%. The extra-axillary sentinel node was the only tumour-positive lymph node in 5% of all patients with tumour-positive sentinel nodes. The postoperative management was changed in 17% of patients with extra-axillary sentinel nodes. Despite the uncertain sensitivity we recommend the pursuit of non-axillary sentinel nodes because of the improved staging, the therapeutic implications and the minimal morbidity.
